# The research progress on the impact of pig gut microbiota on health and production performance

**DOI:** 10.3389/fvets.2025.1564519

**Published:** 2025-03-05

**Authors:** Jing Wang, Tiejin Tong, Changqing Yu, Qiang Wu

**Affiliations:** School of Advanced Agricultural Sciences, Yibin Vocational and Technical College, Yibin, China

**Keywords:** pig gut microbiota, health, production performance, dietary fiber, short-chain fatty acids

## Abstract

Porcine gut microbiota plays a crucial role in the health and productive performance of pigs, influencing nutrient absorption, feed conversion efficiency, and ultimately, production profitability. In addition to being the primary site of digestion, the intestine houses the pig’s largest immune organ, where the microbial community is essential for overall well-being. During the piglet stage, the gut microbiota undergoes a dynamic evolution, gradually adapting to the host environment. This plasticity presents opportunities to intervene and optimize its composition from early stages, enhancing animal health and development. Among the key factors in this process, dietary fiber plays a fundamental role, as its fermentation by the gut microbiota directly affects its composition and functionality, particularly in the distal small intestine, colon, and rectum. The short-chain fatty acids produced during this process not only provide continuous energy to intestinal cells but also regulate immune responses, prevent infections, and contribute to the body’s homeostasis, promoting healthy growth. Despite advancements in understanding host-microbiota interactions, there is still no clear consensus on the optimal balance of gut microbiota or a precise definition of a healthy microbiota. Current research aims to identify the factors that modulate the gastrointestinal microbiota and its physiological and immune functions. Future findings will aid in developing strategies to restore gut homeostasis after external disruptions, such as stress, antibiotic use, or infections, thereby improving productivity, reducing stress-related impacts, and preventing diseases in pig production.

## 1 Importance of gut microbiota

With the deepening research on animal gut microbiota in recent years, it has been confirmed that this microbial community plays a fundamental role in the host’s physiology and health (1). The mammalian gut microbiota is established after birth through continuous interactions with the environment, forming a symbiotic and interdependent system with the organism (2). During the host’s growth and development, the gut microbiota evolves through mutual adaptation and selection processes, consolidating its stability and functionality (3).

The microorganisms residing in the host’s intestine maintain stable colonization due to their interaction with the intestinal wall and various factors influencing their establishment. This colonization begins with aerobic bacteria, followed by facultative anaerobes, and finally, obligate anaerobes (4). The host’s immune response, the presence of intestinal receptors, chyme mobility, nutrient composition, and intestinal pH determine the dynamics of microbial colonization (5).

In pigs, gut microorganisms mainly consist of aerobic bacteria, facultative anaerobes, and obligate anaerobes, with notable genera including *Bifidobacterium*, *Lactococcus*, *Lactobacillus*, *Streptococcus*, and *Enterococcus* (6, 7). Pigs are among the primary species used in biomedical research due to their anatomical and physiological similarities to humans. Their monogastric gastrointestinal tract and gut microbiota make them an ideal model for studies on nutrition, metabolism, and the immune system (8).

Recent studies have demonstrated that porcine gut microbiota significantly influences nutrient digestibility, immune regulation, and protection against opportunistic pathogens, reinforcing its importance in animal production and translational research (9).

The pig’s gut microbiota constitutes a complex and stable ecosystem distributed across different regions of the digestive tract, including the stomach, duodenum, jejunum, ileum, cecum, rectum, and colon. However, this stability can be affected by various factors such as diet type, antibiotic use, and the animal’s physiological state. Therefore, understanding porcine gut microbiota is key to optimizing feed efficiency, improving animal health, and reducing the environmental impact of pig production.

## 2 Composition and functions of the intestinal microbiota

### 2.1 Composition of the porcine intestinal microbiota

The pig’s gastrointestinal tract harbors billions of bacteria and other microorganisms, whose genetic reservoir is 100 times greater than that of the pig’s own genome, which is why it is referred to as the pig’s “second genome.” The colonization and stabilization process of the pig’s intestinal microbiota is dynamic (10).

Previous studies have shown that piglets acquire their early intestinal microbiota through contact with the sow’s birth canal, feces, skin, milk, and surrounding environment (11). However, due to the instability of the early intestinal ecosystem, the composition of microbial communities fluctuates significantly.

After the transition phase of the rearing period, the intestinal microbiota develops a certain level of resistance to external disturbances and enters a stable dynamic equilibrium. The pig’s small intestine is divided into the duodenum, jejunum, and ileum. The duodenum, which contains duodenal glands, is approximately 25–30 cm long. Bile and pancreatic juices flow into the small intestine through the openings of the bile duct and pancreatic duct in the middle of the duodenum (12). There is no clear boundary between the jejunum and ileum. The jejunum accounts for approximately 2/5 of the small intestine and occupies the upper left part of the abdominal cavity, while the ileum makes up about 3/5 and connects to the large intestine at its distal end (13).

Chyme from the stomach is digested in the small intestine with the help of pancreatic juice, bile, and intestinal secretions, breaking down into glucose, fatty acids, amino acids, and other substances. These nutrients are absorbed into the blood and lymph through the small intestine, providing energy for the pig’s growth and development (14). In general, the longer the pig’s small intestine, the better its digestion and absorption of food. The length of the small intestine is influenced by various factors such as age, breed, nutrition, and selection. The pig’s large intestine consists of the cecum, colon, and rectum (2).

The large intestine differs significantly in appearance from the small intestine, having a wider diameter and thinner walls. The large intestine absorbs water, electrolytes, and substances that have not yet been absorbed in the small intestine (15). Although the large intestine’s nutrient absorption capacity is much lower than that of the small intestine, it is primarily the site of fermentation, especially in the cecum (16). Various studies indicate that fermentation in the cecum and proximal colon accounts for more than 90% of the total fermentation in the large intestine. Microbes in the large intestine can ferment and break down fibers and carbohydrates that are not digested in the small intestine, producing large amounts of volatile fatty acids. In different parts of the intestine, microbial distribution exhibits specific regional characteristics (17).

In the small intestine, facultative anaerobes such as *Lactobacillus* and *Bifidobacterium* dominate, primarily involved in preliminary food breakdown and pathogen defense (18). In the colon and cecum, strict anaerobes such as *Clostridia* and *Bacteroides* dominate, mainly fermenting fibers and producing short-chain fatty acids to promote energy supply to the host and intestinal health.

Microorganisms acquired early in life act as initial colonizers and shape the structure of the piglet’s intestinal microbiota (19). Its stability develops in three phases: turbulent, transitional, and stable. The turbulent phase spans from birth to weaning and is characterized by an unstable and constantly changing microbiota (20). Based on their residence time in the intestine, microorganisms are classified as resident or transient. Resident microbes colonize the intestine long-term and maintain stable populations in symbiosis with the host, whereas transient microbes, which are more mobile, include both beneficial and potentially pathogenic bacteria.

The intestinal microbiota is also functionally grouped into beneficial bacteria, opportunistic pathogens, and true pathogens. The latter do not establish themselves permanently but only proliferate when microbial balance is disrupted, causing disease in the animal.

### 2.2 Functions of pig gut microbiota in digestion and nutritional metabolism

Carbohydrates, such as starch and polysaccharides, constitute the primary carbon source for the fermentation carried out by the intestinal microbiota. This process depends on the microorganisms’ ability to synthesize various metabolic enzymes, such as amylase, cellulase, and pectinase, which facilitate the degradation of these compounds. In the porcine ileum, the digestibility of non-starch polysaccharides (NSP) ranges between 15 and 25%; however, after fermentation in the colon, this value significantly increases, reaching between 87 and 90% (21).

Short-chain fatty acids (SCFAs), the products of this fermentation, account for approximately 15 to 24% of the energy supply in adult pigs. Additionally, certain intestinal bacteria, such as *Lactobacilli*, can degrade specific compounds, such as pectin and *β*-glucan, releasing monosaccharides that contribute to energy metabolism (22). In this context, animals store approximately 68% of the energy and 73% of the carbon derived from fermented carbohydrates in the form of volatile fatty acids (VFAs), of with the body can absorb and utilize 94.4–97.3% of the energy (23).

The intestinal microbiota not only participates in the breakdown of dietary and endogenous proteins into short peptides and amino acids but also plays a role in the conversion of free amino acids into polypeptides. Moreover, one of its key functions is vitamin synthesis. It has been demonstrated that germ-free mice, when fed a vitamin K deficient diet, exhibit reduced prothrombin levels and even hemorrhages. In contrast, conventionally colonized mice, under the same dietary conditions, maintain normal prothrombin concentrations and adequate coagulation activity, highlighting the essential role of the microbiota in the production of this vitamin (24). In addition to protein and vitamin metabolism, the intestinal microbiota is involved in the transformation of polyphenols and the bile acid cycle, fundamental processes for host health. The digestibility of non-starch polysaccharides in the pig ileum is 15–25%, and after fermentation by colonic microbiota, it increases to 87–90%, with most of the non-starch polysaccharides being digested by the colonic microbiota (25). The products resulting from this fermentation mainly SCFAs and certain harmful gases.

Subsequently, these SCFAs are converted into VFAs, such as acetic, propionic, and butyric acids, which serve as a key energy source for the pig’s intestine. In this process, acetic acid can be utilized by peripheral tissues, propionic acid is mainly converted into glucose, and butyric acid, apart from serving as an energy source for colonic epithelial cells, has anti-inflammatory effects that contribute to intestinal health (26).

Through fermentative metabolism and biosynthesis, the intestinal microbiota helps compensate for dietary deficiencies, promoting the host’s growth and metabolic balance (27). The gut microbiota, especially members of the *Firmicutes* and *Actinobacteria* phyla, are the main producers of B vitamins. Vitamin K (mainly in its K2 form) is synthesized by specific microorganisms in the intestine and plays a crucial role in blood coagulation and glucose metabolism. K2 not only provides additional microbial support to the host, improving intestinal homeostasis, but also protects the porcine gut microbiota from inefficient transition and utilization. Dietary nitrogen compounds (mainly proteins and polypeptides) are partially hydrolyzed by proteases into peptides and amino acids, which are absorbed by the epithelial cells of the small intestine (28). However, most of them are transported to the large intestine, where they are fermented by the intestine microbiota before being utilized by the host. Additionally, the intestinal microbiota can metabolize other nitrogen sources, such as enzymes secreted by the body, urea, mucin, and shed epithelial cells.

The hydrolysis of dietary proteins by proteases into peptides and amino acids, which are subsequently absorbed by epithelial cells in the small intestine, is considered an efficient mechanism for protein utilization (29). While the microbial community of the small intestine has minimal influence on this absorption process, in the large intestine, the microbiota plays a fundamental role in protein degradation. Both the intestinal microbiota and residual pancreatic proteases present in the colon can break down these nitrogen sources into peptides and amino acids, which are rapidly transformed into short-chain fatty acids to supply energy to the host (30). However, microbial degradation also generates potentially harmful metabolic byproducts, such as amines, ammonia, indoles, and phenolic compounds. Some bacteria such as *Bacteroides*, *Clostridium*, and *Lactobacillus* can produce amines through decarboxylation and break down aromatic amino acids to generate compounds and indoles. Furthermore, the intestinal microbiota not only participates in amino acid absorption and degradation but is also capable of synthesizing them, thus contributing to host metabolism.

### 2.3 The role of the gut microbiota in regulating immune responses in pigs

In recent years, significant progress has been made in understanding the role of the porcine gut microbiota in regulating innate and adaptive immunity. The microbiota influences the pig’s immune system through various mechanisms, such as stimulating the development of immune organs, modulating immune cell functions, and promoting the production of immune factors (31). In innate immunity, its primary contribution lies in strengthening the mucosal barrier and activating immune cells.

One of the key mechanisms of the microbiota is its ability to prevent pathogen colonization, a process influenced by both host factors and the microbiota itself (32). The host contributes to this defense through the secretion of digestive enzymes, electrolytes, mucus, defensins, and secretory IgA. Meanwhile, the microbiota competes with pathogens for epithelial receptors and nutrients, as well as producing antimicrobial substances that inhibit pathogen growth and reproduction (33).

Numerous studies have shown a constant interaction between the gut microbiota and the host’s immune system. While the microbiota promotes immunity development, the immune system, in turn, influences the composition and distribution of the microbiota through effector immune factors (34). One study noted that newborn piglets exclusively rely on maternal milk immunoglobulins to acquire passive immunity. However, when weaned between three and four weeks of age, their immune system is still immature, and at this stage, the gut microbiota plays an essential role in its development (35).

The gut microbiota also modulates T lymphocyte responses, promoting their differentiation according to the type of pathogen present. For instance, infections by intracellular pathogens induce the differentiation of T cells into Th1 cells, producing high levels of interferon, while extracellular parasites stimulate differentiation toward Th2 cells, which secrete IL-4 (interleukin 4) (36). Moreover, segmented filamentous bacteria (SFB) can induce the differentiation of Th17 cells and promote the expression of serum amyloid A (SAA) in the small intestine, stimulating dendritic cells to secrete IL-6 (37).

Species like *Bacteroides fragilis* favor IL-10 production, which, in turn, induces T cells to differentiate into regulatory T cells (Tregs), although this conversion is impaired in IL-10-deficient organisms. Furthermore, one study demonstrated that Tregs are crucial for maintaining immune homeostasis in the host. Additionally, certain species of Firmicutes in the colon induce Treg cell proliferation, strengthening resistance to colitis in murine models. These cells, present in both the small and large intestines, regulate immune responses and minimize adverse reactions to dietary stimuli (38).

Immune recognition of microorganisms is key to immune tolerance. This is because pattern recognition receptors of the immune system, such as the NOD protein family and Toll-like receptors (TLRs), identify conserved microbial structures, allowing differentiation between different bacteria (39). For instance, TLR2 detects peptidoglycan from Gram-positive bacteria, TLR3 recognizes double-stranded RNA from viruses, TLR4 identifies lipopolysaccharide from Gram-negative bacteria, TLR5 detects flagellin, and TLR9 detects bacterial CpG DNA. NOD2, in turn, recognize not only peptidoglycan but also intracellular infections and epithelial invasion by pathogens.

Recent studies have revealed a close relationship between gut microbiota dysbiosis and inflammation. A healthy microbiome, characterized by its diversity and functional stability, contributes to immune balance and the integrity of the intestinal barrier. However, alterations in its composition can trigger inflammatory responses that compromise host health (40).

## 3 Factors influencing changes in the procine gut microbiota

The structure of the gut microbiota is not fixed and changes continuously as the host grows. During this process, the gut microbiota is influenced by various factors such as diet, age, the host’s living environment, antibiotics, and genetic relationships. Diet has a significant impact on the structure of the pig’s gut microbiota. Additionally, the host’s living environment also plays a crucial role in shaping the microbiota, with factors such as weaning, heat stress, feeding practices, and housing conditions all influencing the composition of the gut microbiota.

Weaning has a notable effect on the gut microbiota of piglets (41). Before weaning, the microbiota is dominated by genera such as *Bacteroides*, *Blautia*, *Dorea*, *Escherichia*, and *Clostridium*. After weaning, there is a gradual increase in the genera of *Prevotella* and *Clostridium*, while *Bacteroides* decreases. The increase in *Prevotella* after weaning is likely due to its greater capacity to degrade dietary plant fibers. Heat stress is an important factor affecting the growth performance of pigs during the summer and presents a significant challenge for large-scale pig farming operations. Research by One study has shown that heat stress causes severe damage to the intestinal mucosal structure in pigs (42). This intestinal damage can lead to the invasion of endotoxins, which in turn disrupts the balance of the gut microbiota. Heat stress-induced microbiota imbalances can negatively affect gut health, which, in turn, can lead to poorer growth and performance in pigs.

### 3.1 Dietary factors

The types of food differ according to the pig’s growth stage. For example, during the piglet stage, the diet consists of maternal milk, followed by weaning feed and then growth and fattening food. After 14 days of birth, without any dietary changes, the composition of the gut microbiota remains relatively stable. Over time, although microbial diversity increases, the overall changes are gradual. However, a change in the type of food can significantly alter the composition of the microbiota. For instance, the diversity of the gut microbiota nearly doubles around weaning. During the growth stage (12–16 weeks), feeding with high or low digestibility foods produces notable differences in the pig’s fecal microbiota structure, in the gut microbiota composition of the high digestibility feeding group, the relative abundance of Proteobacteria and Bacteroidetes was significantly lower than that of the low digestibility group (43). Research on commercial pigs during the growth-finishing phase has shown that pigs fed corn/soybean-based diets exhibit higher microbial diversity compared to those fed wheat/barley-based diets.

A study indicated that food is the most influential external factor affecting the composition of the porcine gut microbiota (44). This study also explored the relationship between different food compositions (such as neutral detergent fiber, crude fiber, crude protein, and crude fat) and the porcine gut microbiota, finding that neutral detergent fiber from corn and soybeans has the most significant impact on microbial changes, with increasing proportions of neutral detergent fiber from corn and soybeans, the abundance of beneficial bacteria increased and that of harmful bacteria decreased; Firmicutes and Lactobacillus were the dominant phylum and genus, respectively, and both increased gradually (45). Different types of foods typically have different compositions and nutrient sources, applying various selective pressures on microorganisms, leading to differences in the composition of the gut microbiota. This dietary modulation of the gut microbiota suggests the potential for “dietary therapy” to treating diseases caused by microbial dysbiosis.

### 3.2 Antibiotic factors

The use of antibiotics in livestock production has been a common practice to improve growth, feed efficiency, and animal health. However, in recent years, concerns have emerged about their impact on the gut microbiota of pigs. Prolonged and improper use of these drugs can significantly alter the microbial composition, affecting both the health and performance of the animal. While antibiotics are effective in eliminating or inhibiting bacteria, especially pathogens, they can also reduce microbial diversity by affecting beneficial intestinal bacteria (46).

Recent studies have shown that antibiotics such as chlortetracycline, tylosin, and compound antibiotics can reduce microbiota diversity in pigs. This is because, in addition to eliminating pathogens, they also affect beneficial microorganisms, suppressing sensitive bacteria and favoring the persistence of resistant ones (43).

The effects on gut microbiota do not disappear immediately after the suspension of antibiotic treatment. A study in humans revealed that, after seven days of treatment, resistance genes in the gut microbiota significantly increased and, even after two years, resistance genes continued to be detected in the gut. This suggests that, although antibiotics are essential for disease control, they also promote the proliferation of highly resistant bacteria. Without strict regulation, the gut microbiota could become a reservoir of resistance genes, facilitating their transfer (47).

Excessive use of antibiotics not only promotes the emergence of resistant strains but also contributes to their spread to other animals and humans through the food chain, posing a public health risk. The widespread use of antibiotics in livestock production has been identified as a key factor in the antimicrobial resistance crisis. In response to this issue, China banned their use in animal feed in 2020. Therefore, the search for alternatives such as probiotics and prebiotics to partially or completely replace antibiotics in promoting growth and animal health has become a priority.

### 3.3 Food additives

Probiotics and prebiotics can improve host health by balancing the gut microbiota. Studies have shown that adding *Faecalibacterium* and *Lactobacillus* or increasing prebiotics (such as lactose) can increase the richness and uniformity of the microbiota in piglet feces. After adding the strain *Lactobacillus roschei* ZLR003 to the feed, a decrease in the diversity of the gut microbiota in the jejunum, cecum, and colon was observed, with a notable reduction in the abundance of specific microorganisms such as *Spirillum* species (3, 48). Probiotics can promote the growth of microorganisms that have a positive symbiotic relationship with them, while suppressing the competition with other microbes, thereby altering the composition of the gut microbiota (49). However, this change also carries the risk of disrupting the microbial metabolism within the host, so the effects of probiotics on the host’s microbiota need to be fully evaluated.

### 3.4 Maternal microbiota

The maternal microbiota and the microbiota in the piglet’s growth environment can directly influence the early colonization of the gut microbiota in piglets, and some vertically transmitted microorganisms play an important role in the pig’s growth process (50, 51). The maternal influence on the colonization of occurs during the processes of birth and lactation.

In review. A crossbreeding study found that the influence of the lactating mother on the colonization of the offspring’s gut microbiota was greater than that of the mother who gave birth (52). On the other hand, the gut microbiota of pigs in different environments often has functions that help the host adapt to the environmental characteristics. For example, there is a significant difference in the gut microbiota composition between Tibetan pigs raised at high altitudes and those raised at low altitudes, with high-altitude Tibetan pigs exhibiting a more diverse microbial profile with enhanced capabilities in energy, amino acid, and carbohydrate metabolism, 16S ribosomal meta-analysis results showed significant differences in bacterial diversity and composition between high and low altitude members of the same species, as well as between different species. Acinetobacter, Pseudomonas, and Sphingobacterium were the three most abundant bacterial genera found in high altitude fecal samples of both humans and pigs.

Interestingly, this difference shows a similar trend among high and low-altitude Tibetans. This suggests that, on one hand, the environment selects microorganisms that adapt well to the intestine of humans or animals; on the other hand, these microorganisms help the host adapt better to the environment (53). Some researchers believe that environmental factors are the most significant influence on the host’s gut microbiota after feeding, although other studies have shown that the environment has a less substantial effect on the pig’s gut microbiota compared to factors such as diet and age.

### 3.5 Age factors

In pigs, the influence of age on the gut microbiota may outweigh the effects of maternal influence and host genetics. However, because commercially raised pigs typically receive different nutrient compositions at various stages and are raised in different types of housing environments, the effects of age on the gut microbiota are often confounded by variations in nutrition and housing conditions (54). Postponing the weaning age may therefore favor the development of fiber degrading gut bacteria, conferring the necessary capacity to digest and harvest solid postweaning feed. This results in age-induced changes in the microbiota being either masked or amplified. A study tracked 953 pigs across four different ages to examine age-related shifts in gut microbiota. Firmicutes and Bacteroidetes remained the dominant phyla throughout the study. From 25 to 240 days of age, microbial diversity increased significantly. Principal coordinate analysis revealed notable differences in gut microbial composition between pre-weaning piglets and the other three age groups. Before weaning, piglets exhibited two distinct enterotypes, primarily dominated by Fusobacterium and p-75-a5. In contrast, at 80, 120, and 240 days, Prevotella and Treponema played key roles in shaping the gut microbiota (54, 55).

Additionally, although pigs can live up to 20 years, commercial breeds are usually slaughtered before reaching one year of age, making it difficult to determine how the gut microbiota evolves with aging (56). In a large-scale study of the pig’s life cycle, after isolating the feeding factor, it was found that the diversity index of the pig’s gut microbiota fluctuated significantly during the first 20 weeks, while only slight variations were observed between 2 and 10 years (45). This conclusion deviates significantly from our initial hypothesis, and factors such as the long-term freezing of samples, which were not fully disclosed in the experimental details, complicate the analysis of the cause of this deviation. Therefore, more longitudinal studies focusing on the effect of age are needed.

## 4 Perspectives—opportunities and challenges

With the growing research on the intestinal microbiota of pigs, scientists have gained a profound and comprehensive understanding of its formation, structure, influencing factors, and important roles. The mysterious veil surrounding the “black hole” of the pig’s intestinal microbiota is gradually being unveiled layer by layer, thanks to the rapid development of multi-omics technologies (57). These advances can be categorized into three main technological phases. The first advance was based on traditional isolation and microbiological cultivation techniques, which obtained relatively pure strains under suitable culturing conditions for studies on morphology, classification, and physiological characteristics of the strain (58).

The second advance, starting in the 1980s, involved traditional molecular biology techniques such as DNA fingerprinting and molecular hybridization. These technologies allowed researchers to bypass the limitations of microbial classification and cultivation, enabling the direct detection of changes in microbial community structure or comparative analysis of structural differences between communities (5). However, these techniques had limitations in applicability, sensitivity, and resistance to interference. The third phase began with the advent of high-throughput sequencing technologies, which allowed high-performance DNA sequencing or molecular weight detection in microbial populations, providing identification information at various taxonomic levels, such as genus, species, and strain, further expanding the study of microbiomes through non-cultivation-based methods. With the help of multi-omics technologies, scientists continue to explore beneficial directions of the pig’s intestinal microbiota for health and pig breeding, seeking new health products that could be used as feed additives to improve pig farming.

Currently, the addition of antibiotics to feed remains a critical tool for disease control in China’s pork industry. However, as China moves toward full implementation of regulations banning antibiotics in animal feed, there is an urgent need to find microecological agents that can replace or even surpass antibiotics. The intestinal microbiota, as an essential component of the animal body, has been extensively studied and confirmed to have significant potential in immune responses, and is considered a rich resource that still needs to be fully explored. It can provide new approaches for the prevention and treatment of diseases, although specific molecular mechanisms remain a challenge for scientists worldwide.

The importance of the intestinal microbiota in pig health has become a key focus of animal health research in recent years. The pig’s intestinal microbiota is not only crucial for digestive system function but also plays an important role in immune regulation, disease resistance, metabolic health, and growth performance. A deeper implication of intestinal microbiota research lies in the opportunities and challenges it presents. The opportunity lies in the need for further exploration of the field and a deeper understanding of the mechanisms of interaction between the pig’s intestinal microbiota and the host to better understand the importance of intestinal microbiota. Along with these opportunities are challenges.

Although interventions such as prebiotics, probiotics, and synbiotics have been applied in practice, their variable effects in different pig populations make “precision” intervention a challenge. The development of personalized intervention strategies based on factors such as age, health status, and breeding environment remains a key objective for future research. By properly regulating the intestinal microbiota to improve immunity and health in pigs, it can help reduce pathogen invasion, decrease antibiotic use, and promote more sustainable farming practices. In recent years, with the development of metagenomics, high-throughput sequencing, gene chips, and other technologies, solid technical support has been provided for the continued in-depth study of the intestinal microbiota. As the “second organ” of the body, the intestinal microbiota has great potential to contribute to the prevention and treatment of diseases in both animals and humans. The challenges in intestinal microbiota research mainly stem from its complex ecosystem and individual differences. However, with continuous technological advancements, particularly in precision intervention and personalized treatment, the future promises more precise and efficient regulation of the microbiota. This not only has the potential to improve pig health and production performance but also to provide new approaches to replacing antibiotics and promoting sustainable agriculture.
